# Implementation of a Smart Teaching and Assessment System for High-Quality Cardiopulmonary Resuscitation

**DOI:** 10.3390/diagnostics14100995

**Published:** 2024-05-10

**Authors:** Li-Wen Huang, Yu-Wei Chan, Yu-Tse Tsan, Qi-Xiang Zhang, Wei-Chang Chan, Han-Hsuan Yang

**Affiliations:** 1 Department of Emergency Medicine, Taichung Veterans General Hospital, Taichung 40705, Taiwan; basil2209@gmail.com (L.-W.H.); janyuhjer@gmail.com (Y.-T.T.); fufu110017@gmail.com (W.-C.C.); 2 Department of Computer Science and Information Management, Providence University, Taichung 40301, Taiwan; ywchan@gm.pu.edu.tw; 3 School of Medicine, Chung Shan Medical University, Taichung 40201, Taiwan; 4 Department of Computer Science and Information Engineering, Providence University, Taichung 40301, Taiwan; 5 Everlink Occupational Medicine Clinic, Taichung 40760, Taiwan; b9505019@hotmail.com

**Keywords:** first aid training, cardiopulmonary resuscitation, human pose estimation, deep learning

## Abstract

The purpose of this study is to develop a smart training and assessment system called **SmartCPR**, for teaching and training cardiopulmonary resuscitation (CPR), based on human posture estimation techniques. In this system, trainees can automatically recognize and evaluate whether chest compressions during CPR meet the standard of high-quality CPR by simply using a device such as a smart phone. Through the system, trainees are able to obtain real-time feedback on the quality of compressions so that they can adjust the cycle, depth, frequency, and posture of compressions to meet the standard of high-quality CPR. In addition, the SmartCPR system is convenient for CPR trainers. Trainers can instantly and accurately assess whether the trainee’s compressions meet the standard of high-quality CPR, which reduces the risk of manual assessment errors and also reduces the trainer’s teaching pressures. Therefore, the SmartCPR system developed in this study can be an important tool for CPR teaching and training for physicians, which can provide training and guidance for high-quality CPR maneuvers and enable trainees to become more proficient in CPR and self-training.

## 1. Introduction

Cardiopulmonary resuscitation (CPR) is a fundamental component of initial care for the victim of cardiac arrest. High-quality CPR markedly improves chances of survival and neurological health following out-of-hospital cardiac arrests, emphasizing the essential role of advanced CPR techniques in saving lives and enhancing recovery quality [1,2,3,4,5].

**The key points for high-quality CPR are as follows:**

(1) Proper chest compression position: rescuers should stack their palms, keep their arms straight, ensure an angle of 90° between their arms and the person being compressed, and place the heel of the hand at the midpoint of an imaginary line between the nipples for compressions.
(2) Chest compression depth: the depth of compressions should be between 5 and 6 cm.
(3) Compression rate: at least 100–120 compressions per minute are required.
(4) Chest recoil: ensure the chest fully recoils after each compression.
(5) Minimize interruptions: try to avoid interruptions in compressions, and, if there are any, they must be less than 10 s long [1,6].

Among the standards for high-quality CPR, “chest compression depth” poses the greatest challenge. In the CPR process, overly deep compressions are more likely to cause secondary injuries to the patient’s body. Conversely, an inadequate compression depth can prevent the patient’s heart from pumping sufficient blood to vital organs, adversely affecting the patient’s chances of survival  [1,2,3,4,5]. Additionally, the “chest compression posture” impacts the rescuer’s ability to apply force correctly, which in turn affects the depth and frequency of compressions and ultimately influences the survival rate and brain circulation of the injured or sick. Therefore, the ability to guide CPR trainees accurately and effectively in CPR training, ensuring they adhere to the standards of high-quality CPR regarding “chest compression posture”, “chest compression depth”, and “chest compression frequency”, emerges as a critical aim in promoting high-quality CPR practices.

Enhancing CPR quality is a core element of CPR education and training. Chest compressions guided by CPR feedback devices to ensure adequate rate and depth are beneficial. Multiple studies support using automated real-time feedback devices in CPR training and/or simulations, across both adult and pediatric groups. This approach enhances skill learning, retention and execution in practice scenarios, making the training process more effective [7,8,9,10,11,12,13,14,15,16,17,18,19,20,21].

**Currently, CPR teaching and training methods can be categorized as follows:**

- CPR-qualified instructors teaching on-site: although this method is effective, it is difficult for CPR instructors to accurately determine whether CPR trainees are actually achieving the required 5–6 cm depth of chest compressions and the frequency of at least 100 beats per minute.
- Dummy with embedded sensors and a data monitoring system: A dummy with embedded sensors and a data monitoring function can be used to measure and obtain the compression depth and frequency of the trainee, e.g., Laerdal’s Resusci Anne QCPR (Q-Cardiopulmonary Resuscitation) [22], which can accurately measure and obtain the compression depth and frequency of the CPR trainee. However, the equipment for this system is expensive and not easily available. In addition, the system is unable to determine whether the CPR trainee’s compression position and posture are correct, both of which are critical factors in ensuring high quality CPR.
- Depth-of-field camera and video-based human posture estimation: A depth-of-field camera device (e.g., Microsoft Kinect, Intel RealSense, etc.) extracts data from the human skeleton, analyzes the depth and frequency of chest compressions during CPR, and provides real-time feedback [23]. The disadvantage of this method is that the equipment of this system is costly and not widely distributed compared to general webcams.

**Figure 1:**
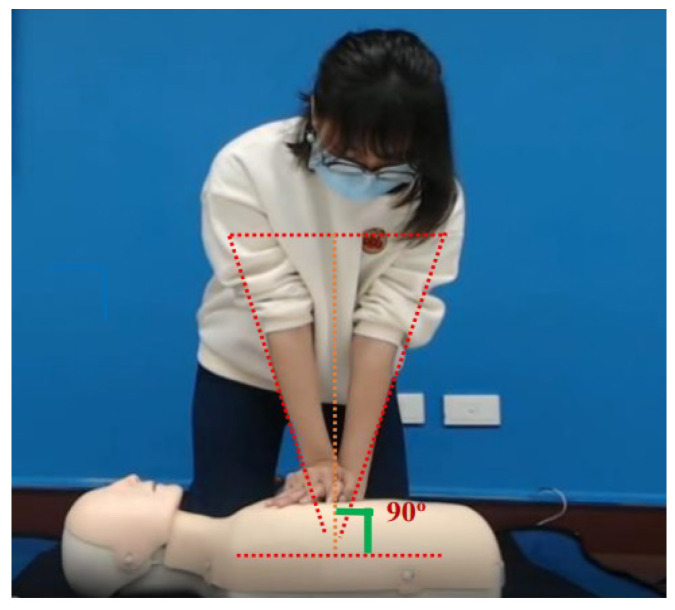
Standard compression pose. The operator’s hands must be folded, the elbows must be straight, and the angle between the elbows and the person being compressed must be 90°.

Effective chest compressions require operators to maintain the correct chest compression posture (CCP). This involves stacking the palms, keeping the elbows straight, and forming a 90° angle between the arms and the patient’s body, as shown in Figure 1 [6]. It has been observed that the degree of elbow extension impacts the depth and frequency of chest compressions, which in turn affects the quality of CPR. Therefore, correcting trainees’ chest compression posture is crucial in CPR training to achieve high-quality CPR.

In this study, we utilized a cost-effective and easily accessible device, such as a cell phone camera, to record the CPR training movements of the trainees. We extracted the key points of the trainee’s skeleton using human posture estimation techniques, without the need for a depth-of-field camera. This approach is more cost-effective and accessible than using devices like Laerdal’s Resusci Anne® QCPR system (QCPR) and large cameras. It specifically addresses the limitations of QCPR, which does not evaluate whether the chest compression posture is appropriate.

We have developed this method to enhance CPR instruction, focusing on promoting the mastery of correct techniques for high-quality CPR. Our design principles and implementations are thoroughly detailed, and we compare them with operational data from QCPR to demonstrate their usability and credibility.

## 2. Related Works

Prior to conducting this study, we first explored the research record of CPR, as well as human posture estimation-related professional fields, including theoretical knowledge, the implementation process, problem handling, and usage. As a result, many previous experiences were drawn upon.

In recent years, studies on the use of the Kinect depth-of-field camera for CPR motion recognition have been presented. In the study of [23], the authors proposed a Kinect-based real-time feedback system for CPR training. In this system, the depth and frequency of chest compressions performed by CPR trainees are displayed on the screen for real-time adjustment. In addition, the authors also proposed using some physical markers placed on the trainee’s hand and chest to obtain Chest Compression Depth(CCD) and Chest Compression Frequency(CCF), which can be used as a reference for trainee training. In [24], the authors proposed a system called RESKIN(RESuscitation KINect), which also utilizes the Kinect skeletal keypoint tracking function to obtain the human skeletal keypoint data in real time. By capturing human motion data during CPR training, the swarm intelligence heuristics algorithm is used to search for target parameters CCD and CCF.

In addition, in a study, the authors proposed a method for evaluating the quality of chest compressions, using OpenPose [25] technology. In the study of [26], the authors proposed utilizing Kinect’s human skeletal keypoint tracking function to capture the skeletal data of human movement in real time. The cosine curve model was used to represent the real-time state of the CPR trainee to calculate the CCD and CCF, and the acquired skeletal keypoints were used to calculate the CPR trainee’s chest compression postural angle and to correct the trainee’s irregular postural position in real time. This study successfully utilized the human motion skeletal data captured by Kinect, combined with the calculated CCF and CCD, to perform standard CPR training and provide real-time feedback to adjust incorrect posture, compression depth, and frequency.

## 3. System Design and Implementation

### 3.1. System Architecture and Processes

**Figure 2:**
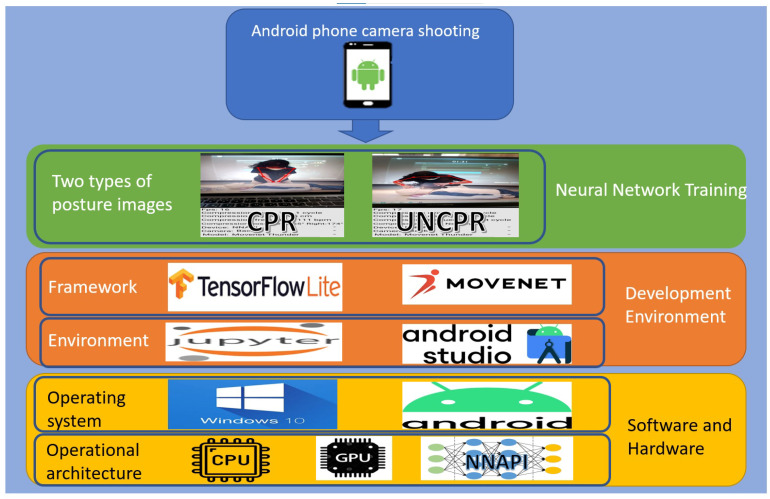
System architecture. The Android phone is used to capture images for pose recognition, utilizing TensorFlow Lite and Movenet frameworks. Trained neural network classifier weights are integrated into the Android Studio environment for development. The hardware includes a computer running Windows 10 and an Android operating system. Along with the CPU and GPU, NNAPI is used on the phone for neural network acceleration.

Next, we introduce the system architecture, which is shown in Figure 2. Firstly, the Android phone is used to take pictures, in order to recognize two different pose images. The main frameworks used are TensorFlow Lite [27] and Movenet [28], and the weight files of the trained neural network classifier are dropped into the Android Studio [29] environment for development. Regarding the required hardware and software, the operating system consists of the computer’s Windows 10 operating system and the Android operating system. In addition to the CPU (Center Processing Unit) and GPU (Graphics Processing Unit), the computing framework on the cell phone can also be accelerated using NNAPI, a neural network application interface computing framework.

**Figure 3:**
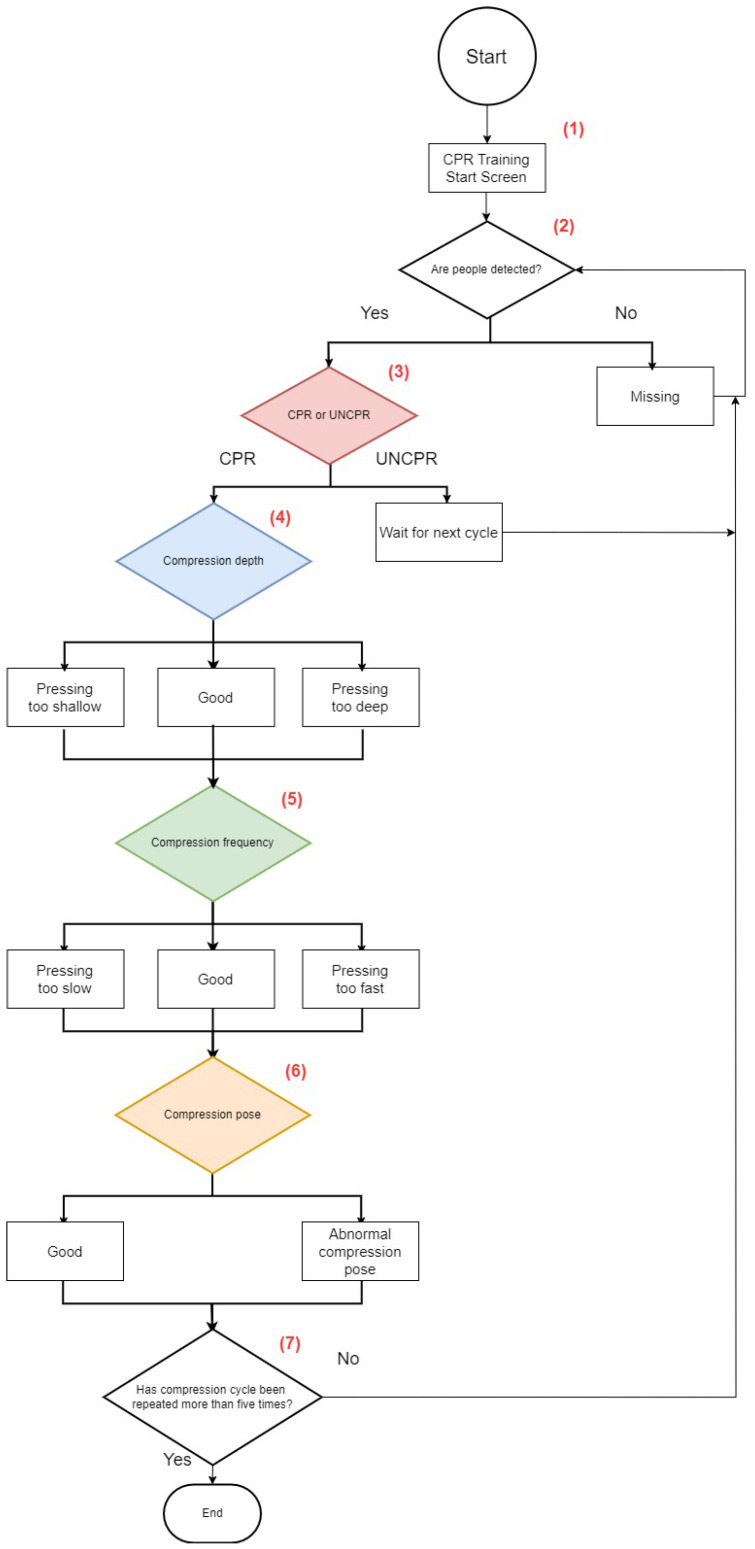
CPRAPP flow chart.

**As shown in Figure 3, the system is designed to assess the quality of chest compressions, using the following steps:**

(1) Activate  Cardiopulmonary Resuscitation APPlication (CPRAPP).
(2) Determine whether people have been detected. If so, continue to the next step; conversely, display “Missing” and then continue to detect.
(3) First, determine whether to press CPR again. If so, continue to the next step; otherwise, display “Next cycle” and return to step (2). If the previous state is UNCPR (Non-CPR state), and the current state is CPR, then the compression cycle will be +1.
(4) The depth of chest compressions is continuously displayed; if the depth of compressions is less than 5 cm, the tone will indicate “Pressing too shallow”. On the other hand, if the compression depth is more than 5 cm, the tone indicates “Pressing too deep”.
(5) Then, the frequency of chest compressions is continuously displayed and calculated; if the pressing speed is less than 100 strokes/min, the voice will indicate “Pressing too slow”; if the pressing speed is more than 120 strokes/min, the voice will indicate “Pressing too fast”.
(6) Next, the compression pose shows whether the compression angles of the left and right hands meet the aforementioned standard of straightening the elbows of the trainee; if they do not, the voice will indicate “Abnormal compression pose”.
(7) Determine whether the compression cycle has been performed more than five times. If the compression cycle has repeated more than five times, then end the detection; otherwise, go back to step (2) and start the whole process again.

### 3.2. System Function Module Introduction

This section describes the main function modules of the system, including the **compression cycle**, **compression depth**, **compression frequency**, and **compression pose** modules.

#### 3.2.1. Compression Cycle Module

**Figure 4:**
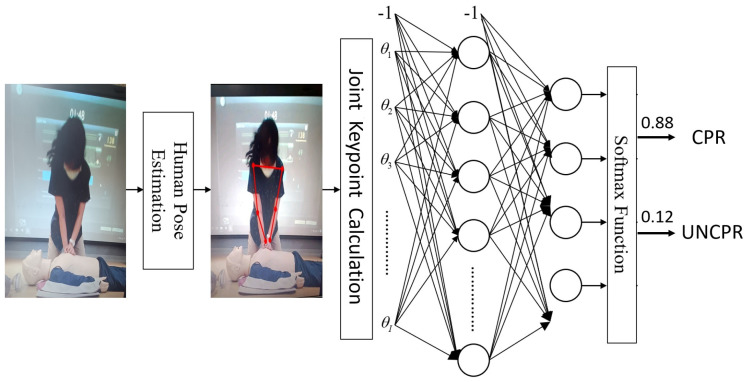
CPR classification model. The neural network classification process distinguishes between the “CPR” and “UNCPR” phases, using the Softmax function to calculate class probabilities.

During the actual CPR procedure, it is not feasible to continuously perform chest compressions. Instead, there is a need to pause after a certain number of compressions to check the patient’s condition or to provide oxygen. Specifically, it is established that every 30 compressions, a check is performed, and this constitutes one cycle. A total of 5 cycles of compressions should be conducted. However, it is challenging to determine the compression phase using programming alone. As shown in Figure 4, we utilize neural network [30,31] classification to discern between the “CPR” and “UNCPR” phases, employing the Softmax function to calculate the probability for each class.

#### 3.2.2. Compression Depth Module

In the Y-coordinate system of Android devices, a higher pixel value corresponds to a lower position, while a lower pixel value corresponds to a higher position. To determine the compression depth, we need to find the lowest position of the left and right wrists. Therefore, we let left_wristY represent the *Y*-value of the left wrist, and right_wristY represent the *Y*-value of the right wrist. The calculation used to obtain the lowest wrist position is as follows:

(1) wristY=max(left_wristY,right_wristY)

To assess the compression depth, we use the following formula:

(2) $$\begin{array}{c} Compressiondepth=\\ \;(wristY2\;-\;initialHeight)/rate \end{array}$$

where wristY2 represents the position of the wrist during the second compression, initial_height represents the initial height before the compression, and *rate* denotes the conversion rate from pixel to actual compression depth.

#### 3.2.3. Compression Frequency Module

In terms of compression frequency, a range of 100 to 120 compressions per minute means that there should be a maximum of two compressions per second (120/60 = 2). This indicates that each compression should ideally take approximately 0.5 s (1/2 = 0.5). On the other hand, the minimum compression rate is 1.667 compressions per second (100/60≈1.667), resulting in an approximate compression time of 0.6 s (1/1.667≈0.6). Therefore, by knowing the time it takes for each compression, we can calculate the compression frequency per minute. The formula for this calculation is as follows:

(3) Compressionfrequency=60/Presstime

For example, if the pressing time is 0.6 s, the compression frequency would be 60/0.6 = 100 bpm (beats per minute).

Next, we will calculate the compression time based on the wrist position (wristY). The algorithm is as follows.

In Algorithm 1, the input is the wrist Y axis position (wristY) detected through the Movenet model. When executing this algorithm, the wristY position is continuously input until the PressTime is determined. The whole loop in this algorithm consists of three states. In state 0, the initialHeight is continuously recorded until wristY starts to move downwards, then it transitions to state 1. In state 1, wristY1 is continuously recorded until wristY starts to move upwards. If the absolute difference between initialHeight and wristY1 is greater than 15.5, then the startTime for pressing is recorded and it transitions to state 2. In state 2, if the absolute difference between wristY and initialHeight is less than five, isReadyForSecondCycle is set to true, and wristY2 is recorded. If wristY2 starts to move upwards and the difference between wristY1 and wristY is less than five, the endTime is recorded, PressTime is calculated, and the loop is exited. Finally, the compression frequency is calculated based on PressTime and returned.
**Algorithm 1:** Compression frequency
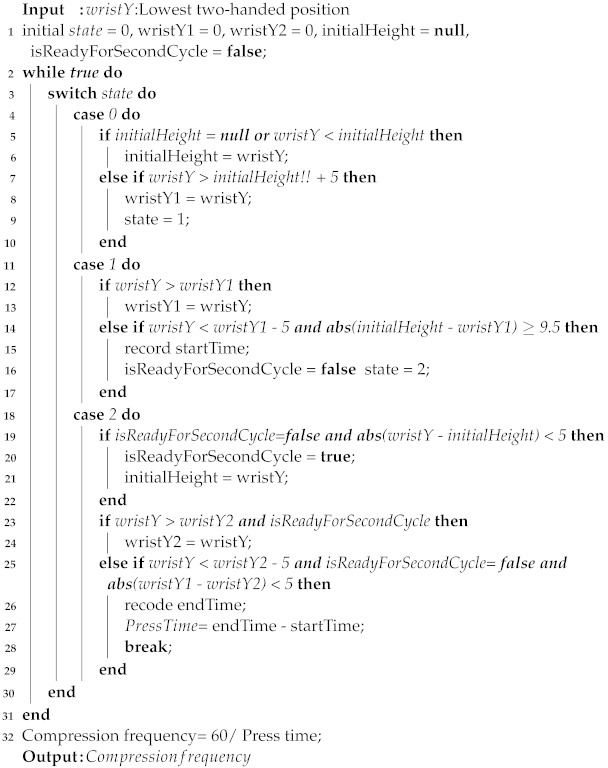


#### 3.2.4. Compression Pose Module

**Figure 5:**
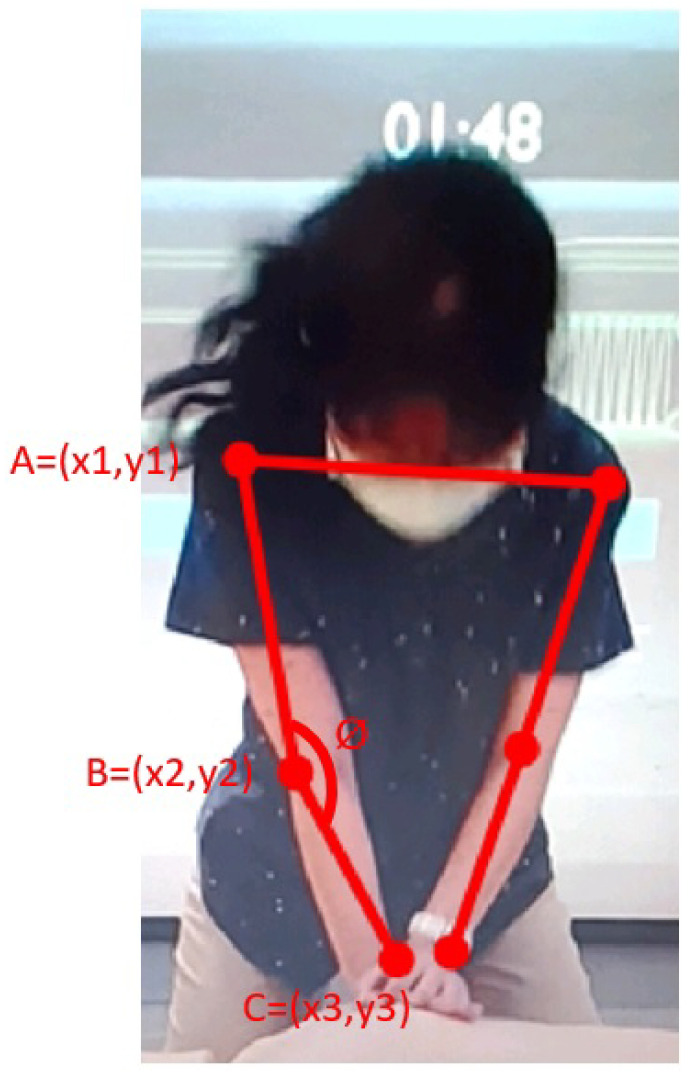
CPR pose. Point A represents the position of the right arm, point B represents the position of the right elbow, and point C represents the position of the right wrist. The distances between these points are denoted by line segments AB (from A to B), BC (from B to C), and AC (from A to C).

In the compression pose, the main focus is on determining the angles of the left and right elbows. If the angle formed by the arms during pressing is greater than 165°, it is considered a correct compression pose. Otherwise, a voice prompt of “Abnormal compression pose” is triggered. As shown in Figure 5, we take the right hand as an example, where point A represents the position of the right arm, point B represents the position of the right elbow, and point C represents the position of the right wrist. The distances between points A and B, B and C, and A and C are represented by the line segments AB, BC, and AC, respectively. We need to calculate the angle ϕ at point B, and we reference the following formula [32] to calculate the angle ϕ:

(4) $$\phi =\frac{BC^{2}+AB^{2}-AC^{2}}{2*BC*AB}$$

## 4. Experiments

### 4.1. Experimental Environment

In this section, we introduce the computer image and software used, and we also introduce the hardware devices on the cell phone. The operating system of the phone is Android, and the computing environment of the device is shown in Table 1.

### 4.2. Experimental CPRAPP Interface

**Figure 6:**
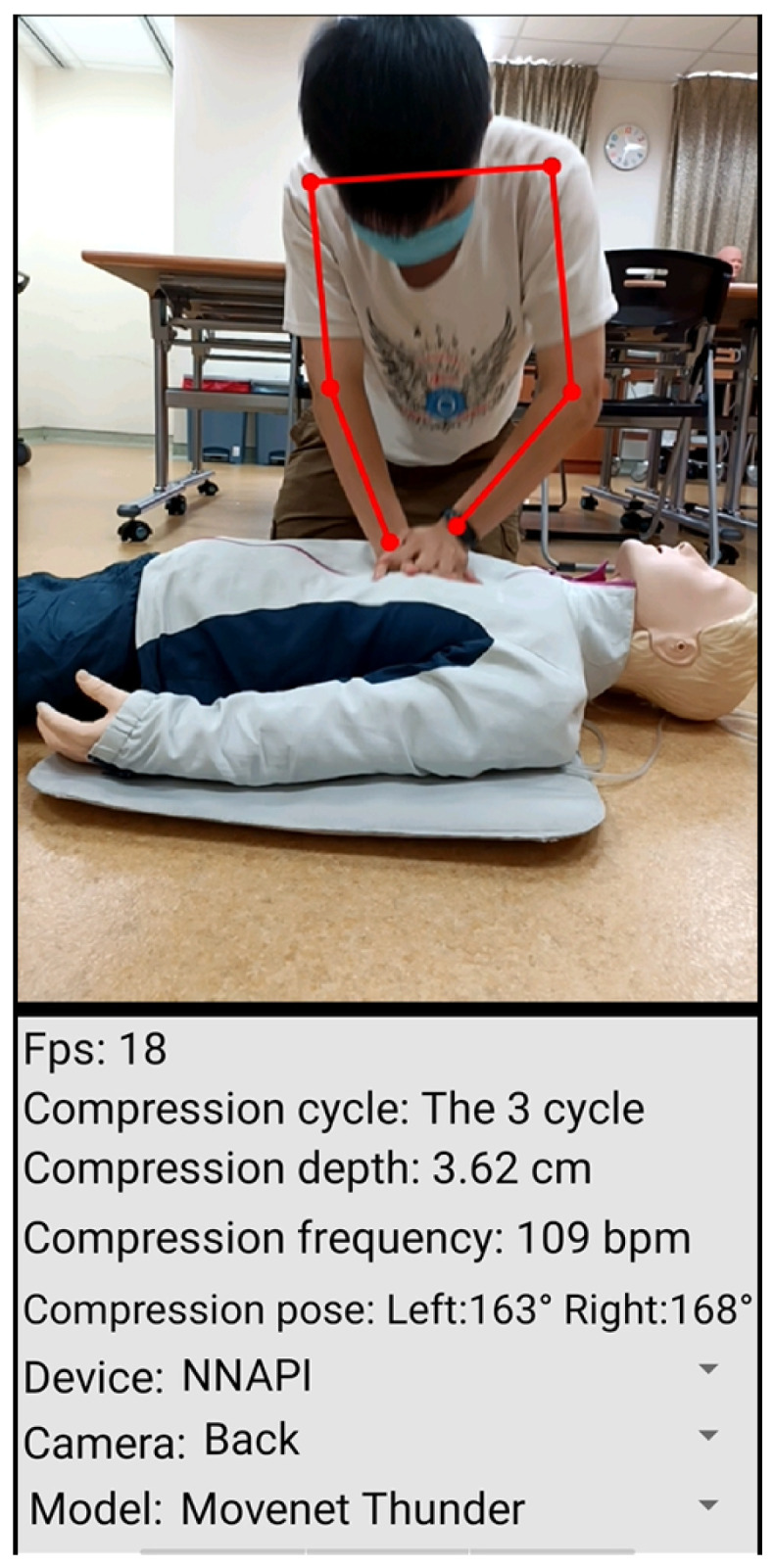
CPRAPP Interface.The upper half shows the detected posture during CPR, and the lower half shows the information obtained during detection.

As shown in Figure 6, in the upper part of the image, there are six red dots, and lines connecting these dots. These points and connections are detected by the Movenet human detection model. The six dots represent the left arm, left elbow, left wrist, right arm, right elbow, and right wrist. These six points are chosen as criteria for evaluation during the CPR procedure.

In the lower part of the image, as shown in Figure 6, we have information about the CPR compression process. Firstly, “Fps” stands for frames per second, indicating the number of frames detected per second. Next, we have “Compression cycle”, which calculates the number of compression cycles during CPR. Each compression cycle consists of 30 compressions, followed by a brief rest period for pulse check, breaths, and other emergency measures. A total of five compression cycles are performed.

1. “Compression depth” indicates the depth measured during compressions; the standard compression depth is 5–6 cm.
2. “Compression frequency” is the number of presses per minute; the normal range is 100– 120 presses per minute. If the pressing frequency is lower than 100 per minute, there will be a voice prompt of “Please press faster”, and, if the pressing frequency is more than 120 per minute, there will be a prompt of “Please press slower”.
3. “Compression pose” refers to the bending angle of the elbows during compression. A normal compression pose requires both hands to be at angles greater than 165°. If the angle of either hand is less than 165°, there will be a voice prompting “Compression pose abnorma”.
4. In the “Device” section, we can choose which device to use for accelerated computing, including CPU, GPU, and NNAPI (Neural Networks Application Programming Interface). Among these, NNAPI has the fastest computing speed, followed by GPU, while CPU has the slowest speed. Regarding the “Camera” section, we can choose to use the rear lens or the front lens for real-time image detection.
5. Finally, “Model” refers to the Movenet model used, and we can choose between Movenet Thunder or Movenet Lightning, with Movenet Thunder being slower but more accurate, and Movenet Lightning being faster but less accurate.

### 4.3. System Experimental Results

**Figure 7:**
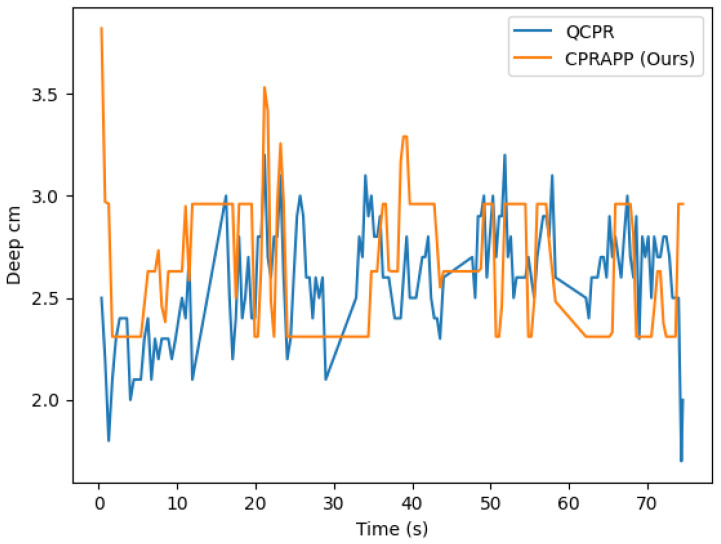
The comparison results between our proposed method, CPRAPP, and QCPR, with respect to the compression depth. The *x*-axis represents press time in seconds, while the *y*-axis represents press depth in centimeters. The blue line indicates the trend of QCPR press depth, and the orange line represents the trend of CPRAPP press depth.

In order to evaluate the performance of our system, we conducted a real-world test, in terms of compression depth, and compared it to the QCPR. If the press depth of our CPRAPP is very close to the QCPR, it means that our CPRAPP has performed well. The compression depth line graph is shown in Figure 7; the horizontal axis is the press time in seconds and the vertical axis is the press depth in centimeters. The blue line is the trend of QCPR press depth and the orange line is the trend of CPRAPP press depth.

In comparing our proposed method (CPRAPP) with QCPR (see Table 2), our mean absolute error (MAE) is 0.32517, which indicates a lower average absolute error than QCPR. Accuracy is assessed by whether the depth error during each compression is less than or equal to 0.5 cm; compressions meeting this criterion are deemed accurate, and those that do not are deemed inaccurate. Our comparison shows an accuracy rate of 84.21052% between QCPR and our CPRAPP, demonstrating a high level of precision in measuring depth error. The *p*-value of 0.05769, being above 0.05, suggests there is insufficient evidence of a significant difference between QCPR and our CPRAPP.

## 5. Conclusions

### 5.1. Discussion

According to the 2020 American Heart Association Guidelines for Cardiopulmonary Resuscitation and Emergency Cardiovascular Care, the use of CPR feedback devices during training is recommended [14]. To deliver a comprehensive and engaging training experience focused on emergency scenarios, including CPR, various high-quality simulation manikins have been specifically developed to provide realistic simulations. Key examples include the Laerdal Resusci Anne® QCPR manikin [22], Ambu® Man Training Manikins [33], TruCorp TruAED (Automated External Defibrillator) [34], and WorldPoint ALS (Amyotrophic lateral sclerosis) Manikins [35]. Additionally, straightforward feedback devices, such as the Physio-Control TrueCPR Coaching Device, CPREzy, Laerdal® CPRmeter, and the Life/form® CPR Metrix Control Box, are employed to enhance learning by providing immediate performance feedback. Numerous comparisons between CPR feedback devices and traditional CPR training methods have shown the positive effects of CPR feedback devices [7,8,9,10,11,12,13,14,15,16,17,18,19,20,21].

CPR training using the Resusci Anne QCPR system developed by Laerdal® has become a mainstream method for real-time feedback device training. The data collected by QCPR can objectively demonstrate the quality of compressions. Thus, besides their primary use in CPR training, QCPR data are also commonly utilized to compare various CPR quality metrics [12,13,36,37,38]. Our research includes comparisons with QCPR to validate its reliability in identifying these metrics as well. While traditional methods that rely on instructor visual assessments lack precision in quantifying speed and depth, QCPR accurately measures these aspects. However, it does not address posture errors or provide immediate feedback [22].

Our system integrates both functions, offering quantification and feedback on compression speed and depth, and it can instantly address posture issues [9,12,13]. In this study, we have developed an intelligent CPR teaching, training, and evaluation system. This system uses MoveNet human pose estimation technology to accurately detect CPR poses, including compression cycle, depth, frequency, and pose, providing real-time feedback and evaluation results. Compared to traditional instruction by an instructor alone, our system provides more precise quantitative data, immediate feedback, and real-time data presentation, enabling instructors to assess trainees’ performance more effectively. The mobile application can be updated instantly for immediate use, thereby enhancing the system’s responsiveness to guideline updates. Additionally, it facilitates immediate corrections, addressing the limitations of QCPR and other simulation manikins. Furthermore, our system is more user-friendly and accessible than QCPR or other simulation manikins [23].

Bystander CPR significantly enhances the chances of survival following an out-of-hospital cardiac arrest [39,40,41]. Lower-cost, more accessible, and effective CPR training is expected to further promote the widespread adoption of CPR among the public. Compared to the Resusci Anne QCPR, our system also judges the accuracy of elbow positioning and provides voice feedback in real time. With its lower cost and the ability to be widely used (detectable, recognizable, and feedback-providable via smartphones), it further facilitates the promotion of correct CPR techniques. The dissemination of this system can also enable more people to learn accurate CPR skills, allowing for more successful patient rescues by buying extra treatment time in emergencies.

Basic life support (BLS) should be administered as soon as possible when cardiac arrest occurs. However, BLS skills often decline rapidly after initial training. Therefore, regular refresher courses and improvements in educational methods for BLS training are essential to enhance skill acquisition and retention [14,42,43,44]. Today, almost everyone owns a mobile phone, underscoring the vast potential of mobile applications, due to their widespread accessibility and flexibility. Our system is currently operational on Android phones, and we aim to extend its use to CPR instruction in the future. This expansion will aid in training both instructors and trainees, utilizing the convenience of mobile apps to disseminate knowledge widely and enable more people to master high-quality CPR techniques.

Virtual reality (VR) simulation has been extensively developed for medical education and various clinical training programs [36,45,46,47,48]. Our system not only retains the existing CPR coaching assistant function but also has the scalability to integrate AI (Artificial Intelligence) time it appears and VR (Virtual Reality), time it appears enhancing more immersive scenario simulations. Moreover, as the program operates on mobile phones, it conveniently leverages the multimedia capabilities of these devices for widespread use, including instructional demonstrations, video replays, and providing teaching and feedback in unmanned environments. It also supports the recording and archiving of repeated practice sessions for comparison. This capability promotes CPR training among healthcare professionals and the general public, thereby reducing the manpower gap in BLS training through frequent retraining. In the future, as standard operation definitions change, parameters can be adjusted promptly and applied in real-time.

### 5.2. Limitations

This study has some research limitations. In the verification results, due to the captured images, women often have their joints obscured by long hair or are under backlit conditions, leading to misjudgments in the results. Currently, the accuracy of deep machine learning is 81%. Follow-up arrangements under regulated conditions, and increasing machine self-learning cases, can effectively improve accuracy.

Our next research study will continue to optimize the app and conduct more comparisons with QCPR to verify the reliability of its data. 

## Figures and Tables

**Table 1:** Computer phone computing environments.

Item	Name	CPU	GPU	OS	DISK	RAM
Computer	ASUSTeK H110-PLUS	Intel (R) Core (TM) i7-6700 CPU 3.40 GHz	Nvidia GeForce GTX 770	Windows 10	1 T	16 G
Android phone	Oppo Reno7 5 G	Dimensity 900 CPU 3.40 GHz	Mali-G68 MC4	Android 12	256 G	8 G

**Table 2:** Comparison compression depth between QCPR and CPRAPP.

Criteria	Explanation	Value
MAE	In the context of QCPR and CPRAPP, the MAE represents the average discrepancy between the compression depths measured by the two methods.	0.32517
Accuracy	Each data point is compared with QCPR. If the compression depth error is <=0.5 cm, it is considered accurate; otherwise, it is considered inaccurate.	84.21052%
*p*-value	A *p*-value of less than 0.05 indicates significant evidence against the null hypothesis, while a *p*-value greater than 0.05 suggests insufficient evidence to reject the null hypothesis.	0.05769

## Data Availability

Authors private data provided.
